# Biallelic mutations in *WRAP53* result in dysfunctional telomeres, Cajal bodies and DNA repair, thereby causing Hoyeraal–Hreidarsson syndrome

**DOI:** 10.1038/s41419-020-2421-4

**Published:** 2020-04-17

**Authors:** Sofie Bergstrand, Stefanie Böhm, Helena Malmgren, Anna Norberg, Mikael Sundin, Ann Nordgren, Marianne Farnebo

**Affiliations:** 1 Department of Bioscience and Nutrition, Karolinska Institutet, Stockholm, Sweden; 2 Department of Cell and Molecular Biology (CMB), Karolinska Institutet, Stockholm, Sweden; 3 Department of Molecular Medicine and Surgery, Center for Molecular Medicine, Karolinska Institutet, Stockholm, Sweden; 4 Clinical Genetics, Karolinska University Hospital, Stockholm, Sweden; 5 Department of Medical Biosciences, Medical and Clinical Genetics, Umeå University, Umeå, Sweden; 6 Section of Hematology, Immunology and HSCT, Astrid Lindgren Children’s Hospital, Karolinska University Hospital, Stockholm, Sweden; 7 Department of Clinical Science, Intervention and Technology, Karolinska Institutet, Stockholm, Sweden

**Keywords:** Telomeres, Cell lineage

## Abstract

Approximately half of all cases of Hoyeraal–Hreidarsson syndrome (HHS), a multisystem disorder characterized by bone marrow failure, developmental defects and very short telomeres, are caused by germline mutations in genes related to telomere biology. However, the varying symptoms and severity of the disease indicate that additional mechanisms are involved. Here, a 3-year-old boy with HHS was found to carry biallelic germline mutations in *WRAP53* (WD40 encoding RNA antisense to p53), that altered two highly conserved amino acids (L283F and R398W) in the WD40 scaffold domain of the protein encoded. WRAP53β (also known as TCAB1 or WDR79) is involved in intracellular trafficking of telomerase, Cajal body functions and DNA repair. We found that both mutations cause destabilization, mislocalization and faulty interactions of WRAP53β, defects linked to misfolding by the TRiC chaperonin complex. Consequently, WRAP53β HHS mutants cannot elongate telomeres, maintain Cajal bodies or repair DNA double-strand breaks. These findings provide a molecular explanation for the pathogenesis underlying WRAP53β-associated HHS and highlight the potential contribution of DNA damage and/or defects in Cajal bodies to the early onset and/or severity of this disease.

## Introduction

The hereditary disorder dyskeratosis congenita (DC), and its most severe form the Hoyeraal–Hreidarsson syndrome (HHS), are associated with severely shortened telomeres and a variety of clinical symptoms, including bone marrow failure, fibrosis in the lung and liver, developmental defects and cancer (mainly hematological and head and neck malignancies), as well as a classical triad of mucocutaneous features (oral leukoplakia, abnormal skin pigmentation, and nail dystrophy)^1^. Patients with HHS also suffer from intrauterine growth retardation, neurological complications and severe immunodeficiency, often resulting in death during childhood^2^.

A central feature of DC and HHS is defective maintenance of telomeres and mutations in one of 11 genes controlling telomere homeostasis, including *DKC1, TERT, RTEL1, PARN, TINF2* (linked to both DC and HHS), *TERC, WRAP53, NOP10, NHP2, CTC1* (linked only to DC), and *ACD* (TPP1 protein, linked only to HHS), have been detected in ~70% of cases with DC and 50% with HHS^3–18^. However, the genetic basis for the remaining cases is still unclear. Moreover, several investigations have revealed that the severity of DC or HHS cannot be explained on the basis of telomere length alone^19^. For example, patients with mutations in the core components of telomerase (i.e., the reverse transcriptase TERT and TERC RNA) exhibit milder disease, with onset during adolescence or early adulthood. In contrast, those with mutations in genes with additional functions, including *DKC1, PARN*, and *RTEL1*, demonstrate more severe forms of DC or HHS, with early onset and short life expectancy. Furthermore, the length of telomeres in some patients with severe clinical symptoms is actually normal^20,21^ while some individuals, belonging to families with a history of HHS and who carry relevant mutations, exhibit very short telomeres, but no clinical manifestations^18^. Such observations indicate that perturbations other than those in telomeres are involved in the etiology of DC and HHS.

One such additional multifunctional gene in which inherited mutations result in severe DC is *WRAP53*^14,22^, originally identified in our laboratory as an antisense gene to the p53 tumor suppressor^23^. *WRAP53* codes for both a regulator RNA (WRAP53α) that stabilizes p53 RNA and a protein of 75 kD (WRAP53β, also referred to as TCAB1 and WDR79) involved in telomerase trafficking, maintenance of Cajal bodies and DNA repair^24–26^. The WD40 domain of WRAP53β serves as a scaffold for interactions between multiple factors and appears to be essential to its function. Indeed, the five mutations in WRAP53β observed to date in three DC patients (i.e., F164L/R398W; H376Y/G435R and R298W/R298W) are all located within this domain^14,27^, four of these are reported to cause misfolding of the WRAP53β protein that attenuates its interactions with telomerase, thereby preventing trafficking of telomerase to telomeres^28^.

In addition to binding telomerase, the WD40 domain of WRAP53β scaffolds interactions between the SMN and coilin proteins, required for their localization to Cajal bodies and for structural maintenance of these organelles^26^. This WD40 domain also scaffold interactions between repair factors that are necessary for their recruitment to and repair of DNA breaks^29^. Thus, dysfunctional interactions and/or related processes might contribute to the severity of clinical symptoms caused by mutations in WRAP53β.

Here, we demonstrate that germline mutations in *WRAP53* are involved in the etiology of HHS, showing that L283F and R398W alterations in WRAP53β disrupt its interactions not only with telomerase but also with Cajal body and DNA repair factors. Consequently, in addition to the presence of shortened telomeres, maintenance of Cajal bodies and repair of DNA double-strand breaks are attenuated when WRAP53β is mutated. We propose that defects in functions related to Cajal bodies and incomplete repair of DNA breaks, in combination with progressive shortening of telomeres, underlie the severe phenotypes of DC and HHS, associated with disruptive mutations in WRAP53β.

## Results

### Clinical characterization

**Fig. 1:**
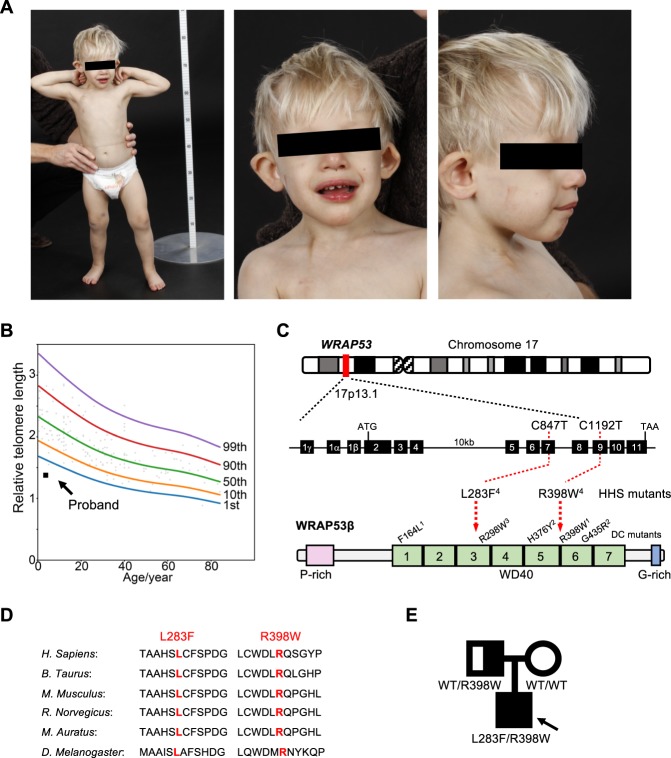
Identification of missense mutations in *WRAP53* in a patient with HHS. **a** At an age of 2.7 years, the proband demonstrated microcephaly, short stature, broad gait, fine blond hair and dysmorphic features (including strabismus, epicanthal folds, cup-shaped protruding overfolded ears, a depressed nasal tip and widely spaced teeth). **b** Analysis of telomere lengths by quantitative PCR in peripheral blood leukocytes collected from the proband at this same age (solid square). The reference relative telomere length (RTL) were determined from telomere length analysis of blood leukocytes from 173 healthy subjects (0–84 years of age; open circles). The curves shown depict the first, 10th, 50th, 90th, and 99th normal percentiles at each age. **c** Schematic illustration of the *WRAP53* gene, the protein encoded and the positions of the mutations detected in the proband. The DC mutations in WRAP53β reported previously are also marked with the superscripts indicating mutations that occur in the same patient. Note: Exon numbering is based on the GenBank sequence DQ431240, i.e., the separation of exon 1β and 2 by an intron. In the reference sequence NM 018081 this intron is included resulting in different exon numbering. **d** Conservation of the sites of HHS mutations (marked in red) among species. **e** Pedigree of the family carrying autosomal recessive HHS and mutations in *WRAP53*. The arrow indicates the proband. The heterozygous carrier (the father, half-filled symbol), compound heterozygous carrier (the proband, filled symbol), and wild-type individual (the mother, non-filled symbol) are shown.

Born following IVF, the male proband was the first child of healthy, non-consanguineous parents with no history of bone marrow failure. Because of severe intrauterine growth restriction (IUGH) (reflected in the birth weight of 1242 g, length 39 cm, head circumference 27 cm (all −3.5 SD, apgar scores 10, 10, 10), acute Caesarean section was performed at 33 weeks of gestational age. Clinical features consistent with HHS were debuted during his early years of life, including microcephaly, cerebellar hypoplasia, developmental delay, delayed psychomotor development, progressive bone marrow failure, gastrointestinal complications, liver fibrosis, intellectual disability, and retinal changes (summarized in Table 1). This boy was short, with hypotonia and dysmorphic facial features. Other than pale skin with darker areas around the eyes, neither skin abnormalities nor dystrophic nails, often observed in patients with DC, were detected (Fig. 1a). His hearing and cardiac function appeared normal.

**Table 1:** Characteristics of the proband and his parents.

Individual	Gender	Age when the study was performed (yrs)	Diagnosis	Clinical features/abnormalities	Telomere length	Genotype
Proband	Male	6	HHS	Intrauterine growth restriction (IUGH) *Gastrointestinal complications* Necrotizing enterocolitis (NEC) Volvulus (50% of his small intestine was removed) Digestive tract anomalies and eating difficulties (i.e., oesophageal strictures, varices, swallowing difficulties and severe hemorrhages) *Neurological complications* Microcephaly Cerebellar hypoplasia Atrophy of the vermis cerebelli Cortical malformation Poor myelinization Delayed psychomotor development (i.e., broad gait, mild intellectual disability, no speech) *Bone marrow failure and immunodeficiency* Thrombocytopenia Erythropenia Recurrent infections *Other complications* Fibrosis (grade 3 out of 4) and malformation of the liver Dilated intrahepatic choledochus Elevated blood levels of transaminases Retinal abnormalities and vitreous body hemorrhaging Dysmorphic features of the face and body (i.e., strabismus, epicanthal folds, slightly deep-set eyes, dark gray eyes, cup-shaped protruding overfolded ears, depressed nasal tip, fine blond hair, widely spaced teeth, full lips, everted upper lip vermilion, fragile, fast growing nails, short stature and hypotonia) Pale skin	<1st percentile	WRAP53^L283F^ /WRAP53^R398W^
Father	Male	45	Healthy	None	Not determined	WRAP53^WT^ /WRAP53^R398W^
Mother	Female	44	Healthy	None	Not determined	WRAP53^WT^ /WRAP53^WT^

At 21 months of age the proband required transfusions once every other week, due to his thrombocytopenia and erythropenia. Hematopoietic stem cell transplantation was counterindicated by the severity of his disease and, instead, he received androgen therapy. Six years old at the time of this study, he was refractory to infusion of thrombocytes, which he only received when bleeding. He suffered several life-threatening esophageal bleedings. The proband had no speech but communicated with signs and was fed through a gastric tube.

### Genetic analysis and identification of mutations in *WRAP53*

Despite his delayed development and dysmorphic features, comparative genomic hybridization (CGH) revealed normal copy numbers of chromosomes. In agreement with his HHS-like symptoms, analysis of his peripheral blood leukocytes revealed very short telomeres (below the first percentile in comparison to those of healthy controls of the same age) (Fig. 1b). Targeted sequencing of genes known to be related to DC/HHS (i.e., *TERC*, *TINF2*, and *DKC1*) did not, however, reveal any mutations. Thus, the proband had classical symptoms of HHS, including extremely short telomeres, but no mutations in genes known to be associated with this disease.

To identify potential underlying mutations, whole-exome sequencing of DNA from the boy and both of his parents was performed. The proband carried two heterozygous missense mutations in the *WRAP53* gene (Gene ID 55135): nucleotides c.847C>T and c.1192C>T, corresponding to p.Leu283Phe (L283F) and p.Arg398Trp (R398W) in the resulting protein (Fig. 1c). Heterozygous mutations resulting in R398W has been detected previously in one case of DC, but then in combination with a F164L mutation^14^, while L283F has not been reported earlier.

The predicted structure of the WRAP53β protein includes an unstructured N-terminal region (including a proline-rich section), a domain with seven WD40 repeats, followed by a short C-terminal extension containing a glycine-rich region (Fig. 1c)^30^. Both L283 and R398 are located within the WD40 domain of WRAP53β and are strictly conserved in all species examined to date, including mammals and flies (Fig. 1d).

The healthy father harbored a single *WRAP53* mutation (R398W), but did not demonstrate any manifestations of disease, indicative of autosomal recessive inheritance (Fig. 1e). The healthy mother had two wild-type alleles in peripheral blood, indicating that the second mutation in the proband (L283F) had occurred de novo (although maternal gonadal mosaicism cannot be excluded). The healthy younger sister of the proband was not tested.

### Mutations in WRAP53β impair its localization to the nucleus and Cajal bodies as well as its structural functions in Cajal body

**Fig. 2:**
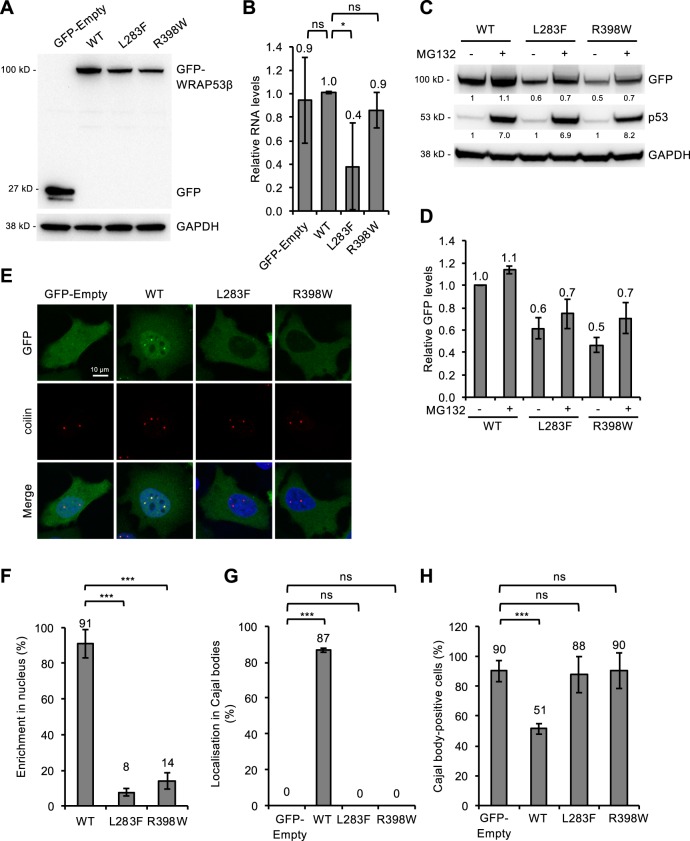
WRAP53β mutants associated with HHS are expressed at reduced levels and with an altered localization, disrupting the structure of Cajal bodies. **a** HeLa cells were transfected with a vector encoding: EGFP only (GFP-Empty), or EGFP-tagged wild-type (WT) or mutant (L283F or R398W) WRAP53β for 24 h and thereafter subjected to western blot for GFP and GAPDH. **b** Quantitative PCR analysis of the levels of mRNA encoding EGFP alone (GFP-Empty), EGFP-tagged wild-type (WT) or mutant (L283F or R398W) WRAP53β in HeLa cells 24 h post-transfection. Primers targeting the GFP sequence were utilized to avoid detection of endogenous WRAP53β. The values shown represent the RNA levels normalized to β-actin and relative to the levels of GFP-WT WRAP53β. **c** HeLa cells were transfected with a vector encoding: EGFP-tagged wild-type (WT) or mutant (L283F or R398W) WRAP53β for 16 h followed by addition of the protease inhibitor MG132 (10 μM) for another 8 h, as indicated. GFP, p53, and GAPDH were detected by western blot. The levels of p53, a protein with rapid turnover via the proteasome, was used as a control for proteasome inhibition by MG132. The numbers in black represent densitometric quantification of each protein normalized to GAPDH and relative to the levels of wild-type WRAP53β (for the GFP proteins) or p53 (for the p53 protein) in cells not treated with MG132 (first lane). **d** The same densitometric numbers as shown in **c**, including error bars. Again, the values represent the GFP levels relative to the levels of wild-type WRAP53β. **e** Immunofluorescent detection of the GFP-tagged WRAP53β proteins indicated in HeLa cells 24 h post-transfection. The Cajal bodies were visualized by immunostaining for coilin and nuclei stained blue with DAPI in all cases. **f** Quantification of cells as shown in **e** exhibiting nuclear enrichment of the GFP-tagged wild-type or mutant WRAP53β. The bars indicate the percentage of 100 GFP-positive cells in each experiment in which the nuclear signal was at least as intense as the signal in the cytoplasm (i.e., with a nuclear/cytoplasmic signal intensity ratio ≥1). **g** Quantification of cells as shown in **e** in which GFP accumulated in Cajal bodies. The bars indicate the percentage of 100 cells in each experiment that stained for both GFP and Cajal bodies and in which these bodies were enriched for GFP. **h** Quantification of cells as shown in **e** containing Cajal bodies. The bars indicate the percentage of 100 GFP-positive cells whose nuclei also contained Cajal bodies (i.e., formation of coilin foci). In all cases, the values are means ± s.d. (the error bars) (*n* = 3). *p < 0.05, **p < 0.01, ***p < 0.001, ns (not significant) as determined by Student’s *t*-test.

To explore how the L283F and R398W changes might influence the behavior of the WRAP53β protein, these mutations were individually incorporated into a vector encoding EGFP-WRAP53β that was then expressed transiently in HeLa cells. Western blotting of whole-cell lysates revealed lower levels of both mutant proteins than of the wild-type transfected WRAP53β (Fig. 2a). In addition, the RNA levels of L283F, but not R398W, were also reduced (Fig. 2b), indicating that the stability of both WRAP53β RNA (at least for L283F) and the corresponding protein are reduced by these mutations. The efficiency of transfection (40–60% of cells, data not shown) was similar in all cases and thus could not explain the differences observed.

To examine whether accelerated proteasomal degradation of the WRAP53β mutants could be an underlying cause of their lower levels, cells expressing these proteins were treated with MG132, a proteasome inhibitor for 8 h before the protein levels were measured. Although both mutants showed a slightly higher stabilization following MG132 compared with wild-type protein (1.2-fold for L283F, 1.5-fold for R398W and 1.1-fold for wild-type), their levels were not restored to those of the wild-type WRAP53β (Fig. 2c, d). Together, this indicate that RNA destabilization is the main cause of the lower L283F protein levels, while in the case of R398W, whose RNA levels were normal, an increased rate of protein degradation in combination with a yet unknown factor appear to underlie the reduced protein levels.

Examination by immunofluorescence revealed that the wild-type WRAP53β protein localizes in the cytoplasm and nucleus and is enriched in nuclear Cajal bodies, as reported previously^14,24,26^. In contrast, the mutant forms were localized to a lesser extent in the nucleus and absent from the Cajal bodies (Fig. 2e–g).

Proper assembly of WRAP53β in Cajal bodies is essential for their maintenance, as demonstrated by the findings that either loss or overexpression of WRAP53β causes these organelles to collapse, after which they cannot re-form^24,26,31,32^. Accordingly, overexpression of exogenous wild-type WRAP53β here led to the disappearance and/or prevented the formation of Cajal bodies in approximately half of the cells transfected (Fig. 2h). Interestingly, overexpression of the mutant forms of WRAP53β or of GFP itself produced no such effect (Fig. 2h), indicating that the structural function of WRAP53β in connection with Cajal bodies is attenuated by the mutations associated with HHS, probably due to the inability of the mutant proteins to localize in these organelles. Together, these findings demonstrate that the mutations in WRAP53β associated with HHS render the protein unstable, cause mislocalization of the protein, and perturb its structural functions in Cajal bodies.

### Mislocalization of mutant WRAP53β is due to alterations in their interactions with components of the Cajal body and TRiC chaperonin complex

**Fig. 3:**
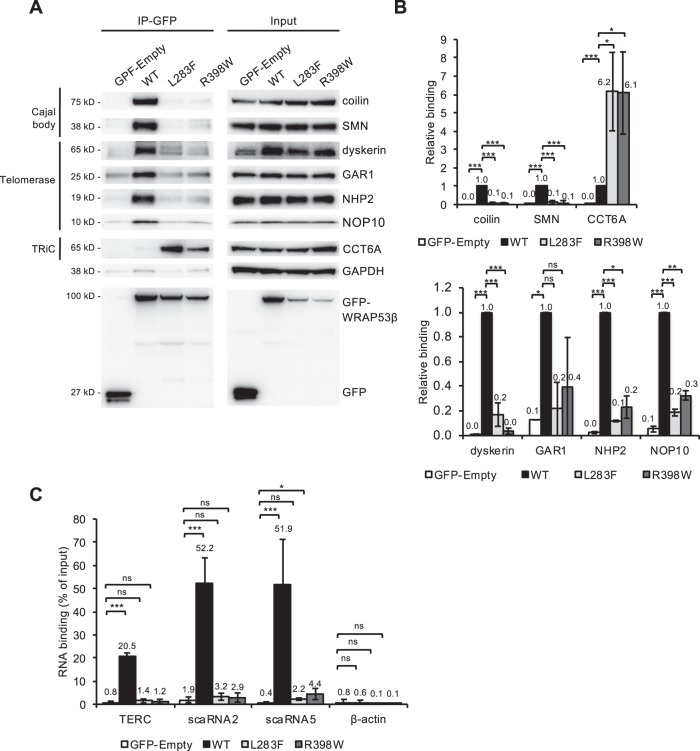
HHS mutants of WRAP53β interact less with components of Cajal bodies and telomerase but more with those of the TRiC complex. **a** HeLa cells were transfected with the GFP proteins indicated for 24 h and then subjected to immunoprecipitation with a GFP antibody, followed by immunoblotting. **b** Densitometric quantifications of western blots as shown in **a**. The bars represent the levels of co-precipitated protein normalized to the levels of the corresponding immunoprecipitated GFP protein itself and then relative to the value obtained in wild-type WRAP53β (i.e., levels of (co-precipitated protein/immunoprecipitated protein itself)/(co-precipitated protein by wild-type WRAP53β/immunoprecipitated wild-type WRAP53β itself)). The values are means ± s.d. (the error bars) (*n* = 3). *p < 0.05, **p < 0.01, ***p < 0.001, ns (not significant) as determined by Student’s *t*-test. **c** HeLa cells were treated as in **a**, followed by purification of RNA and assessment of the levels of co-precipitated RNA employing qPCR. The values show the amount of co-precipitated RNA as a percentage of input with the error bars depicting the s.d. (*n* = 3 for scaRNA5 or 4 for TERC, scaRNA2 and actin). *p < 0.05, ***p < 0.001, ns (not significant) as determined by Student’s *t*-test.

Localization of WRAP53β to Cajal bodies requires its interaction with coilin and SMN^26^. While immunoprecipitation of wild-type WRAP53β efficiently co-precipitated both of these proteins, neither WRAP53β L283F nor R398W did (Fig. 3a, b), indicating that loss of interaction with coilin and SMN underlies the attenuated accumulation of mutant WRAP53β in Cajal bodies.

Impaired folding of these mutant proteins by the TRiC chaperonin complex might also contribute to their mislocalization, as well as to reduced stability, as observed previously for DC mutants of WRAP53β^28^. Immunoprecipitation, revealed an enhanced interaction of both mutant forms of WRAP53β with the TRiC chaperonin component CCT6A in comparison to the wild-type protein (Fig. 3a, b). Since WRAP53β is only released from TRiC upon proper folding^28^, it appears like the folding of the mutants is impaired. In addition, this enhanced interaction sequesters WRAP53β mutants in the cytoplasm, preventing its entry into the nucleus (Fig. 2e, f). We conclude that HHS-associated mutations in WRAP53β cause misfolding of the protein and disrupt its interactions with SMN and coilin, thereby preventing its entry into the nucleus and localization in Cajal bodies.

### Mutations in WRAP53β disrupt its interaction with components of telomerase and scaRNAs

In addition to its structural role in Cajal bodies, WRAP53β targets telomerase and scaRNAs to these organelles. Immunoprecipitation of GFP-WRAP53β followed by western blotting or RT-PCR confirmed that the wild-type protein co-precipitates with components of the telomerase complex (including dyskerin, NHP2, NOP10, GAR1 and TERC) (Fig. 3a, b), as well as with various scaRNAs (including those with C/D boxes (i.e., scaRNA2) and H/ACA boxes (i.e., scaRNA5 and TERC)) (Fig. 3c). In contrast, neither HHS-mutant of WRAP53β co-precipitated telomerase components or scaRNAs (Fig. 3a–c). The fact that disruption of the WRAP53β-TERC interaction prevents the targeting of telomerase to telomeres provides a mechanistic explanation for the severely reduced telomere length in our proband (Fig. 1b).

The loss of binding between mutant WRAP53β and components of Cajal bodies and telomerase was almost complete and greater than the reduced expression of the mutants (Fig. 3b). Moreover, interaction was also lost under conditions when the levels of the mutants were similar as wild-type (data not shown). Furthermore, the enhanced interaction between mutant WRAP53β and CCT6A demonstrates that the extent of interaction does not necessarily reflect the level of the protein.

### HHS-associated mutations abrogate the involvement of WRAP53β in repair of DNA double-strand breaks

**Fig. 4:**
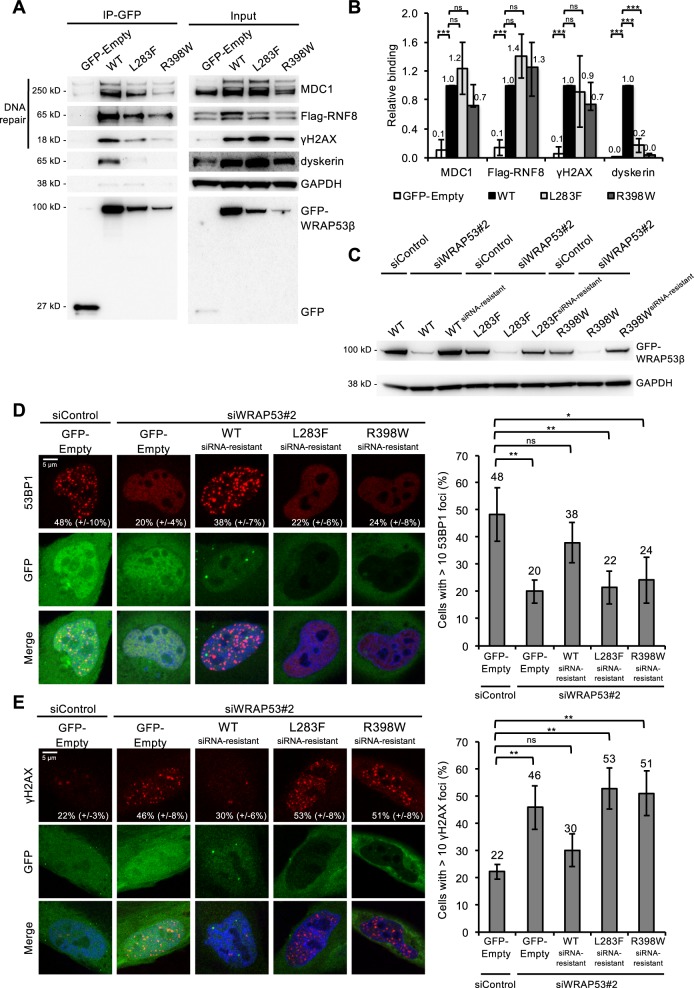
HHS-associated WRAP53β mutations attenuate repair of DNA double-strand breaks and lead to accumulation of DNA damage. **a** HeLa cells were transfected with the GFP plasmids indicated and Flag-RNF8 for 24 h; irradiated with  2 Gy; left to recover for 1 h; and then subjected to immunoprecipitation with a GFP antibody, followed by immunoblotting. **b** Densitometric quantifications of western blots as shown in **a**. The bars represent the levels of co-precipitated protein normalized to levels of the corresponding immunoprecipitated GFP protein itself, and then relative to the value obtained in wild-type WRAP53β. The values are means ± s.d. (the error bars) (*n* = 3). *p < 0.05, **p < 0.01, ***p < 0.001, ns (not significant) as determined by Student’s *t*-test. **c**, **d** HeLa cells were transfected with siControl or siWRAP53#2 oligonucleotides for 24 h; followed by transfection with GFP-Empty or GFP-WRAP53β^siRNA resistant^ plasmids for another 24 h; irradiated with 2 Gy; and 1 h later harvested for either western blotting using the antibodies indicated (**c**) or immunostaining for 53BP1 (**d**). The graph in **d** shows the percentage of 100 GFP-transfected cells in each experiment whose nuclei contained ≥10 53BP1 foci with the error bars depicting the s.d. (*n* = 4). **e** HeLa cells were transfected with siRNA for 8 h; followed by transfection with the GFP plasmids indicated for another 16 h; irradiated with 2 Gy; and after 24 h of recovery, immunostained for γH2AX. The graph shows the percentage of 100 GFP-transfected cells in each experiment whose nuclei contained ≥10 γH2AX foci with the error bars depicting the s.d. (*n* = 3). *p < 0.05, **p < 0.01, ns (not significant) as determined by Student’s *t*-test.

The mutant forms of WRAP53β retained the capacity to bind the DNA repair proteins RNF8, MDC1 and γH2AX, although somewhat less extensively, particularly in the case of γH2AX (Fig. 4a, b). This finding is similar to previous observations on the DC-associated mutants of WRAP53β (F164L, H376Y, R398W and G435R)^29,33^. We conclude that interactions between WRAP53β and DNA repair proteins are less dependent on structure/folding of WRAP53β than its interaction with Cajal body factors and components of telomerase, but may, instead, depend on the linear sequence.

When DNA damage occurs, WRAP53β targets the repair factor RNF8 to the lesions and facilitates subsequent recruitment of downstream repair factors, including 53BP1^29,34^. Following siRNA depletion of the endogenous wild-type WRAP53β the assembly of 53BP1 in repair foci is reduced and we next explored whether HHS mutants of WRAP53β could reverse this loss of 53BP1 assembly on damaged chromatin. When constructs encoding siRNA-resistant forms of wild-type, L283F, or R398W WRAP53β were introduced into cells depleted of the endogenous protein, only the wild-type could restore the formation of 53BP1 foci following induction of DNA damage by IR (Fig. 4c, d).

The presence of residual γH2AX foci 24 h after damaging DNA with IR is a sign of defective repair. As observed in connection with the formation of repair foci, re-introduction of the L283F or R398W mutant following knockdown of endogenous WRAP53β could not resolve residual γH2AX foci, whereas the wild-type protein did (Fig. 4e).

**Fig. 5:**
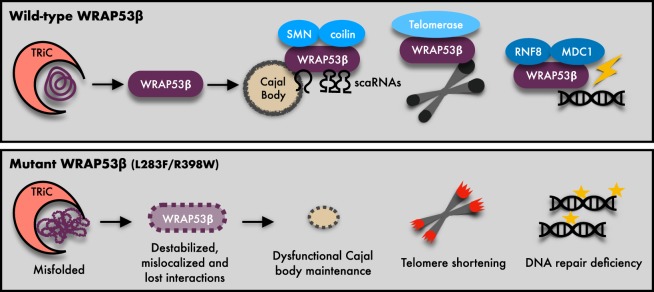
Model of defects caused by HHS mutations in *WRAP53*. Schematic illustrations of the normal folding and functions of wild-type (WT) WRAP53β and molecular defects and functional consequences of the HHS mutations. *WRAP53* mutations results in misfolding and destabilization of the WRAP53β protein and impair the binding to and trafficking of partner proteins and RNAs to correct cellular sites. Consequently, this perturbs maintenance of Cajal bodies, shortens telomeres and impairs DNA repair, thereby causing HHS.

In summary, our findings demonstrate that HHS-linked mutations in *WRAP53* lead to destabilization, mislocalization and impairment of its function in Cajal bodies, at telomeres and in DNA repair. These combined deficiencies contribute to the multiple symptoms and severity of HHS (Fig. 5).

## Discussion

Here, we show for the first time that biallelic mutations in *WRAP53* result in HHS. The missense mutations identified (L283F and R398W) are both located in the WD40 scaffolding domain of the WRAP53β protein and affect amino acids that are highly conserved across species. By scaffolding appropriate interactions, WRAP53β plays essential roles in localizing various factors to the nuclear organelles known as Cajal bodies, to telomeres and to DNA double-strand breaks and is thus important in connection with the regulation of nuclear architecture, telomere elongation and DNA repair^24,26,29,31^.

Although the explanation for the wide range in severity of DC (including development of HHS) and age of onset remain unclear, the specific genes involved and the mode of inheritance provide part of the answer. In the case of WRAP53β, inherited biallelic mutations affecting its WD40 domain have previously been reported in three patients and linked to DC, including the R398W mutation^14,22,27^. All patients exhibited at early ages severe symptoms of classic DC, including bone marrow failure, very short telomeres and the triad of skin pigmentation, oral leukoplakia and nail dysplasia. The 30-year-old male reported by Shao et al. had short stature, liver cirrhosis, portal hypertension, splenomegaly, marked decreased platelet levels, esophageal varices, and recurrent gastrointestinal hemorrhages, and the 13-year-old male reported by Zhong and Batista et al. was diagnosed with esophageal web and narrowing, fusion of the kidneys and pervasive developmental disorder. It is possible that certain mutations, including the novel L283F mutation described here, are more detrimental than others. Indeed, mRNAs containing the c.847C>T mutation (encoding L283F) are expressed at much lower levels than those containing c.1192C>T (encoding R398W), indicating RNA destabilization by the former. However, the functional consequences of L283F and R398W, as examined here, were similar in both cases.

WRAP53β is normally present in the cytoplasm and, at the same time highly enriched in nuclear Cajal bodies. We demonstrate here that the L283F and R398W mutants of WRAP53β are unstable, cannot enter the nucleus properly and do not accumulate in Cajal bodies. Our finding that the interaction between the mutant forms of WRAP53β and the cytoplasmic TRiC chaperonin complex is enhanced, indicates that these mutant proteins are misfolded and therefore cannot be released from this chaperone. These mutants are also unable to bind coilin and SMN. Since TRiC-mediated folding and binding to coilin and SMN are required for accumulation of WRAP53β in Cajal bodies, these observations explain why accumulation of the mutant proteins does not occur^26,28^.

The inability of mutant WRAP53β to accumulate in Cajal bodies and/or bind known interaction partners prevented various functions of this protein at this site. First, high overexpression of the mutant forms did not result in the collapse of Cajal bodies and/or inability of this organelle to form, in contrast to high overexpression of the wild-type protein^26,32^. Second, the mutant proteins no longer interacted with SMN, coilin, scaRNAs or telomerase, interactions known to be required for the localization of these factors to Cajal bodies^24–26^. In agreement with such impaired trafficking of telomerase, the telomeres in the proband with HHS were extremely short.

Our proband exhibited a variety of neurological abnormalities, including microcephaly, cerebellar hypoplasia, poor myelinization, delayed psychomotor development, and broad gait. Indeed, underdevelopment of the brain is a key clinical manifestation of HHS and a requirement for definitive diagnosis^35^. Interestingly, loss of WRAP53β-mediated trafficking of SMN has been observed in patients with spinal muscular atrophy^26^, a severe neurodegenerative disorder characterized by progressive degeneration of α-motor neurons in the spinal cord, resulting in paralysis and in severe cases, death. Accordingly, depletion of WRAP53β in *Drosophila* and *Caenorhabditis elegance* resulted in defective locomotion and the death of motor neurons, consistent with the spinal muscular atrophy elicited by depletion of SMN^36^. Mechanistically, expression of SMN in the WRAP53β-deficient cells was reduced and restoration of the level of SMN reversed the defects in locomotion in both species. Importantly, overexpression of WRAP53β also eliminated the defects in locomotion linked to SMN-deficiency in both *Drosophila* and *Caenorhabditis elegance*, demonstrating conserved collaboration between WRAP53β and SMN in this context. Thus, unrelated to telomeres, defective WRAP53β-SMN interplay may cause the neurological complications of WRAP53β-associated HHS. Furthermore, localization of SMN and scaRNAs in Cajal bodies is important for modification and maturation of spliceosomal small nuclear RNAs and dysfunction of this process and, ultimately, of splicing could contribute to the diverse clinical manifestations of HHS and DC.

To date, little evidence links defects in DNA repair to HHS. With the exception of the RTEL1 helicase that suppresses homologous recombination^37–39^, none of the other proteins encoded by HHS-related genes (*DKC1, TERT, ACD, PARN*, and *TINF2*) has been directly implicated in DNA repair. In contrast, WRAP53β has an established role in the repair of DNA double-strand breaks, scaffolding interactions between the repair factors MDC1 and RNF8 at these lesions and thereby promoting subsequent repair by homologous recombination and non-homologous end-joining^29,32,40^. Our current data reveal that although HHS mutations in WRAP53β reduce its interactions with MDC1, RNF8 and γH2AX to only a moderate extent, they significantly impair assembly of the downstream 53BP1 repair factor at DNA lesions, attenuate the repair of IR-induced damage, and result in accumulation of DNA damage.

Interestingly, WRAP53β organizes and resolves persistent DNA damage in neurons^41^, such accumulation of damage is believed to contribute to neurodegeneration, again indicating a telomeric-independent function of WRAP53β in connection with brain homeostasis. Together, these results indicate that WRAP53β-mediated defects in DNA repair in the brain and in other tissues may represent a novel contribution to the severity of HHS and DC in general.

In summary, our present findings reveal that compound heterozygous mutations in *WRAP53* cause HHS by disrupting telomere elongation, DNA repair and functions associated with SMN. These combined deficiencies may explain the complex manifestations of this disease in patients. Our observations also highlight the potential contributions of defects in DNA repair and SMN to other cases of HHS and DC cases, where specific underlying mechanisms have yet to be fully elucidated.

## Materials and methods

### Inclusion of the patient and ethical considerations

The patient was referred to the Department of Clinical Genetics, Karolinska University Hospital in Stockholm, Sweden. This study was performed in accordance with the Declaration of Helsinki and pre-approved by the local ethical board in Stockholm. Informed consent was obtained from the proband and his parents in accordance with local ethical guidelines, including publication of the photos of the proband.

### Array comparative genomic hybridization

To detect variations in copy number, the 180 K custom array (Oxford Gene Technology, Oxford, UK) and a 244k catalogue array (Agilent Technologies, Santa Clara, USA) was utilized. All experiments were performed in accordance with the manufacturer’s recommendations, with minor modifications only. Scanning and the computational procedures have been described previously^42^.

### Sequence analysis genes known to be related to DC

Targeted sequencing of *TERC*, *TINF2* (exon 6), and *DKC1* (exon 3, 4, and 11) was performed at Queen Mary University (London).

### Whole-exome capture and resequencing

DNA from the proband and both parents, isolated from peripheral blood employing standard procedures, was sent for exome sequencing by Oxford Gene Technology (OGT; Begbroke Science Park, Oxfordshire, UK). These samples were subjected to exome capturing using Agilent SureSelect All Exon (v4). Whole-exome sequencing (WES) was performed on an Illumina HiSeq 2500 instrument and 20x coverage was achieved for 90% of the targeted sequence. These sequences were mapped and compared with the reference sequence (UCSC hg 19) in the published human genome. Data were analyzed with software provided by Oxford Gene Technology. Variants detected were selected for further analysis on the basis of de novo and autosomal recessive (homozygous and compound heterozygous) inheritance. The presence of suspected pathogenic variants was verified by Sanger sequencing (primers and PCR conditions available on request).

### Determining telomere length

DNA extracted from peripheral blood leukocytes was analyzed for relative telomere length (RTL) by quantitative PCR^43^, with minor modifications as described earlier^44^. In brief, triplicates were subjected to separate telomere (T) and single copy gene (S) reactions in an ABI 7900HT instrument (Applied Biosystems) on two separate occasions. T/S values were calculated as 2^−ΔCt^, where ΔCt = Ct_(T)_ − Ct_(S)_. The RTL value was obtained by dividing the sample T/S value by the T/S of a reference DNA from a cell line (CCRF-CEM) included in all runs. The RTL value of the sample was compared with that of 173 normal controls (age 0–84 years). The generalized additive model included in the GAMLSS R package was used to calculate centile curves. A best-fit model was obtained by applying the Generalised Akaike information criterion, which revealed the Box-Cox Cole and Green distribution to be optimal. “Very short” telomeres were defined as these shorter than the first percentile of the age-matched normal controls.

### Cell lines, culture conditions, and transfections

HeLa cells (ATCC) were cultured in low-glucose Dulbecco’s modified Eagle medium (Gibco, ThermoFisher Scientific) supplemented with 10% fetal bovine serum (Gibco, ThermoFisher Scientific) and 1% Penicillin/Streptomycin (Gibco, ThermoFisher Scientific) at 37 °C in a humidified incubator under 5% CO_2_. Plasmid transfections were performed using Lipofectamine 2000 (Life Technologies, ThermoFisher Scientific) in accordance with the manufacturer’s recommendations and utilizing GFP-Empty, GFP-WRAP53β, GFP-WRAP53β_L283F, GFP-WRAP53β_R398W, and Flag-RNF8 expression vectors. Mutations in the WRAP53β plasmids were generated with the QuikChange II XL Site-Directed Mutagenesis kit (Agilent) using the following primers: WRAP53_L283F_For: GGCAGCCCATTCGTTCTGCTTCTCCCC, WRAP53_L283F_Rev: GGGGAGAAGCAGAACGAATGGGCTGCC, WRAP53_R398W_For: CTGTGCTGGGATCTCTGGCAGTCTGGTTACC, WRAP53_R398W_Rev: GGTAACCAGACTGCCAGAGATCCCAGCACAG, WRAP53_siW2res_For: GCAAACGGGAGTCTCTCTGAAGAAGAAGC, WRAP53_siW2res_Rev: GCTTCTTCTTCAGAGAGACTCCCGTTTGC. siRNA (15 nM) targeting WRAP53β (SI00388948, Qiagen) or the scramble control (1027280, Qiagen) were transfected into the cells for 48 h employing the HiPerfect transfection reagent (Qiagen) in accordance with the manufacturer’s recommendations. The protease inhibitor MG132 (Merck) was used at a concentration of 10 μM for 8 h.

### Ionizing radiation

γ-Irradiation was performed with the X-Ray Irradiator CIX2 (X-strahl) at settings of 195 kV and 10 mA, a focus-to-specimen distance (FSD) of 40 cm, and a 3 mm Aluminum filter. The dose was ~1.3 Gy/min.

### Immunofluorescent microscopy

Cells grown on sterilized cover slips were fixed with 4% paraformaldehyde for 15 min at room temperature; permeabilized with 0.1% Triton X-100 for 5 min at room temperature; and then incubated for 1 h in blocking buffer (2% BSA, 5% glycerol, 0.2% Tween20, 0.1% NaN_3_). The cover slips were subsequently incubated with primary antibodies for 1 h at room temperature, followed by 30 min with the secondary antibody, both diluted in blocking buffer. After mounting with Vectashield medium containing DAPI (Vector Laboratories, Bionordika), images were acquired with an LSM700 confocal microscope (Zeiss) mounted on a Zeiss Axio observer.Z1 equipped with Plan-Apochromat ×63/1.4 oil immersion lenses and subsequently processed utilizing the Zen 2012 Black software (Zeiss).

### Western blotting

Cells were harvested, washed and lysed in ice-cold lysis buffer (50 mM Tris-HCL, pH 8, 150 mM NaCl, 1% NP-40, 1% protease inhibitor cocktail) for 30 min on ice. The lysates were cleared by centrifugation at 14,000 rpm for 10 min at 4 °C and the protein concentration determined with the Bradford assay (Bio-Rad). Proteins were resolved on 10% NuPAGE^®^ Bis-Tris or 3–8% Tris-Acetate precast gels (Life Technologies, ThermoFisher Scientific) and transferred onto nitrocellulose membranes. Western blotting was performed by standard procedures and the blots developed using SuperSignal West Femto Maximum Sensitivity Substrate (ThermoFisher Scientific). Protein levels were quantified by image densitometry of western blot images using ImageJ.

### Antibodies

The following antibodies against the human proteins were utilized for immunofluorescent staining and western blotting: rabbit α-CCT6A (HPA045576, Atlas Antibodies), mouse α-coilin (sc-56298, Santa Cruz Biotechnology), rabbit α-coilin (sc-32860, Santa Cruz Biotechnology), rabbit α-DKC1 (sc-48794, Santa Cruz Biotechnology), mouse α-GAPDH (sc-47724, Santa Cruz Biotechnology), rabbit α-GAR1 (11711-1-AP, ProteinTech), rabbit α-GFP (ab290, Abcam), rabbit α-IgG (12-370, Millipore, Merck), mouse α-MDC1 (ab50003, Abcam), mouse α-NHP2 (sc-398430, Santa Cruz Biotechnology), rabbit α-NOP10 (ab134902, Abcam), mouse α-RNF8 (sc-271462, Santa Cruz Biotechnology), mouse α-SMN (610647, BD Biosciences), rabbit α-53BP1 (NB100- 904, Novus Biologicals, Bio-Techne), mouse α-γH2AX (05-636, Millipore), rabbit α-γH2AX (2577, Cell Signaling, Bionordika). The secondary antibodies used were goat α-rabbit HRP-conjugated (cat. no. 7074, Cell Signaling), horse α-mouse HRP-conjugated (cat. no. 7076, Cell Signaling), donkey α-mouse Alexa Fluor 594 (cat. no. A21203, Invitrogen), and donkey α-rabbit Alexa Fluor 594 (cat. no. A21207, Invitrogen).

### Immunoprecipitation

Cells were washed twice with ice-cold PBS, scraped of the plates and pelleted by centrifugation. The pellet thus obtained was treated with ice-cold lysis buffer (50 mM Tris-HCL, pH 8, 150 mM NaCl, 1% NP-40, 1% protease inhibitor cocktail) for 15 min on ice, followed by sonication at high intensity for 5 min (30 s ON/30 s OFF) in a Bioruptor UCD-200 (Diagenode). The resulting lysate was then centrifuged at 6000 rpm for 5 min at 4 °C and 1 mg supernatant protein incubated with 10 μl Dynabeads^TM^ Protein G (Invitrogen) and 1 μg GFP antibody overnight at 4 °C with rotation. Thereafter, the beads were washed four times with lysis buffer and prepared for western blotting.

### RNA Immunoprecipitation

Cell lysates were prepared for overnight immunoprecipitation as described above. The beads were washed twice in lysis buffer and then twice in RIPA buffer. RNA was extracted with TRIzol^®^ (Life Technologies, ThermoFisher Scientific), 1-bromo-3-chloropropane (Sigma-Aldrich), and the RNeasy Mini Kit (Qiagen), following the manufacturer’s instructions.

### qPCR analysis

cDNA was generated with SuperScript IV reverse transcriptase (Invitrogen, ThermoFisher Scientific), random hexamer primers (ThermoFisher Scientific), 10 nM dNTPs Mix (ThermoFisher Scientific) and RNaseOUT (Invitrogen, ThermoFisher Scientific) in accordance with manufacturer’s instructions. Enrichment of specific RNA was determined by qPCR in a 7500 Fast Real-Time PCR System (Applied Biosystems, ThermoFisher Scientific) on 96-well fast PCR Plates (Sarstedt) using Fast SYBR™ Green Master Mix (Applied Biosystems, ThermoFisher Scientific) and the following primers, TERC For: GTGGTGGCCATTTTTTGTCTAAC, TERC Rev: TGCTCTAGAATGAACGGTGGAA, scaRNA2 For: GGTTGGAGCGTGTTAGGC, scaRNA2 Rev: GGAGGAGACCTTTTCATTTCG, scaRNA5 For: TGAATGTCACGGTCCCTTTGT, scaRNA5 Rev: AGCTGCTCCATGATCCCATAC, β-actin For: AGAGCTACGAGCTGCCTGAC, β-actin Rev: AGCACTGTGTTGGCGTACAG, GFP For: CTCCTGCCCGACAACCAC, GFP Rev: TCACGAACTCCAGCAGGAC.

### Quantification and statistical analysis

Quantitative immunofluorescence was performed in at least three independent experiments. Statistical details and the numbers of cells analyzed are indicated in the figure legends. Statistical analysis with non-paired two-tailed Student’s *t*-tests was carried out in the Microsoft Excel 16.16.12 software.

## Supplementary information

Marked-up version

Athor contribution

Reproducibility
