# Supplementary material for: Biallelic mutations in WRAP53 result in dysfunctional telomeres, Cajal bodies and DNA repair, thereby causing Hoyeraal–Hreidarsson syndrome
Source: Cell Death Dis. 2020 Apr 17;11(4):238. doi: 10.1038/s41419-020-2421-4 (PMC7165179; doi:10.1038/s41419-020-2421-4)
Supplement: Supplementary file 1 — Marked-up version [file 41419_2020_2421_MOESM1_ESM.docx]

# Biallelic mutations in *WRAP53* result in dysfunctional telomeres, Cajal bodies and DNA repair, thereby causing Hoyeraal-Hreidarsson syndrome

### Sofie Bergstrand^1^, Stefanie Böhm^2^, Helena Malmgren^3,4^Anna Norberg^5^, Mikael Sundin^6,7^ Ann Nordgren^3,4,#^ and Marianne Farnebo^1,2,#,^ *

^1^Department of Bioscience and Nutrition, Karolinska Institutet, Stockholm, Sweden.

^2^Department of Cell and Molecular Biology (CMB), Karolinska Institutet, Stockholm, Sweden.

^3^Department of Molecular Medicine and Surgery, Center for Molecular Medicine,

Karolinska Institutet, Stockholm, Sweden

^4^Clinical Genetics, Karolinska University Hospital, Clinical Genetics, Stockholm, Sweden

^5^Department of Medical Biosciences, Medical and Clinical Genetics, Umeå University, Umeå, Sweden

^6^Section of Hematology, Immunology and HSCT, Astrid Lindgren Children's Hospital, Karolinska University Hospital, Stockholm, Sweden.

^7^Department of Clinical Science, Intervention and Technology, Karolinska Institutet, Stockholm, Sweden.

^#^ These authors contributed equally to this work

Corresponding author: [marianne.farnebo@ki.se](mailto:marianne.farnebo@ki.se)

Running title: Mutations in *WRAP53* cause HHS

**Abstract**

Approximately half of all cases of Hoyeraal-Hreidarsson syndrome (HHS), a multisystem disorder characterized by bone marrow failure, developmental defects and very short telomeres, are caused by germline mutations in genes related to telomere biology. However, the varying symptoms and severity of the disease indicate that additional mechanisms are involved. Here, a 3-year-old boy with HHS was found to carry biallelic germline mutations in *WRAP53* (WD40 encoding RNA antisense to p53), that altered two highly conserved amino acids (L283F and R398W) in the WD40 scaffold domain of the protein encoded. WRAP53β (also known as TCAB1 or WDR79) is involved in intracellular trafficking of telomerase, Cajal body functions and DNA repair. We found that both mutations cause destabilization, mislocalization and faulty interactions of WRAP53β, defects linked to misfolding by the TRiC chaperonin complex. Consequently, WRAP53β HHS-mutants cannot elongate telomeres, maintain Cajal bodies or repair DNA double-strand breaks. These findings provide a molecular explanation for the pathogenesis underlying WRAP53β-associated HHS and highlight the potential contribution of DNA damage and/or defects in Cajal bodies to the early onset and/or severity of this disease.

**Keywords:** WRAP53, Hoyerhaal-Hreidarsson syndrome, telomerase, Cajal body, DNA repair, SMN, 53BP1

**Introduction**

The hereditary disorder dyskeratosis congenita (DC), and its most severe form the Hoyeraal–Hreidarsson syndrome (HHS), are associated with severely shortened telomeres and a variety of clinical symptoms, including bone marrow failure, fibrosis in the lung and liver, developmental defects and cancer (mainly hematological and head and neck malignancies), as well as a classical triad of mucocutaneous features (oral leukoplakia, abnormal skin pigmentation and nail dystrophy) ^1^. Patients with HHS also suffer from intrauterine growth retardation, neurological complications and severe immunodeficiency, often resulting in death during childhood ^2^.

A central feature of DC and HHS is defective maintenance of telomeres and mutations in one of 11 genes controlling telomere homeostasis, including *DKC1, TERT, RTEL1, PARN, TINF2* (linked to both DC and HHS), *TERC, WRAP53, NOP10, NHP2, CTC1* (linked only to DC) and *ACD* (TPP1 protein, linked only to HHS), have been detected in approximately 70% of cases with DC and 50% with HHS ^3-18^. However, the genetic basis for the remaining cases is still unclear. Moreover, several investigations have revealed that the severity of DC or HHS cannot be explained on the basis of telomere length alone ^19^. For example, patients with mutations in the core components of telomerase (i.e., the reverse transcriptase TERT and TERC RNA) exhibit milder disease, with onset during adolescence or early adulthood. In contrast, those with mutations in genes with additional functions, including *DKC1, PARN* and *RTEL1,* demonstrate more severe forms of DC or HHS, with early onset and short life expectancy. Furthermore, the length of telomeres in some patients with severe clinical symptoms is actually normal ^20,21^ while some individuals, belonging to families with a history of HHS and who carry relevant mutations, exhibit very short telomeres, but no clinical manifestations (Kocak 2014). Such observations indicate that perturbations other than those in telomeres are involved in the etiology of DC and HHS.

One such additional multifunctional gene in which inherited mutations result in severe DC is *WRAP53* ^14,22^, originally identified in our laboratory as an antisense gene to the p53 tumor suppressor ^23^. *WRAP53* codes for both a regulator RNA (WRAP53α) that stabilizes p53 RNA and a protein of 75 kD (WRAP53β, also referred to as TCAB1 and WDR79) involved in telomerase trafficking, maintenance of Cajal bodies and DNA repair ^24-26^. The WD40 domain of WRAP53β serves as a scaffold for interactions between multiple factors and appears to be essential to its function. Indeed, the five mutations in WRAP53β observed to date in three DC patients (i.e., F164L/R398W; H376Y/G435R and R298W/R298W) are all located within this domain ^14,27^, four of these are reported to cause misfolding of the WRAP53β protein that attenuates its interactions with telomerase, thereby preventing trafficking of telomerase to telomeres ^28^.

In addition to binding telomerase, the WD40 domain of WRAP53β scaffolds interactions between the SMN and coilin proteins, required for their localization to Cajal bodies and for structural maintenance of these organelles ^26^. This WD40 domain also scaffold interactions between repair factors that are necessary for their recruitment to and repair of DNA breaks ^29^. Thus, dysfunctional interactions and/or related processes might contribute to the severity of clinical symptoms caused by mutations in WRAP53β.

Here, we demonstrate that germline mutations in *WRAP53* are involved in the etiology of HHS, showing that L283F and R398W alterations in WRAP53β disrupt its interactions not only with telomerase but also with Cajal body and DNA repair factors. Consequently, in addition to the presence of shortened telomeres, maintenance of Cajal bodies and repair of DNA double-strand breaks are attenuated when WRAP53β is mutated. We propose that defects in functions related to Cajal bodies and incomplete repair of DNA breaks, in combination with progressive shortening of telomeres, underlie the severe phenotypes of DC and HHS, associated with disruptive mutations in WRAP53β.

**Results**

### **Clinical characterization**

Born following IVF, the male proband was the first child of healthy, non-consanguineous parents with no history of bone marrow failure. Because of severe intrauterine growth restriction (IUGH) (reflected in the birth weight of 1242 g, length 39 cm, head circumference 27 cm (all -3.5 SD, apgar scores 10, 10, 10), acute Caesarean section was performed at 33 weeks of gestational age. Clinical features consistent with HHS were debuted during his early years of life, including microcephaly, cerebellar hypoplasia, developmental delay, delayed psychomotor development, progressive bone marrow failure, gastrointestinal complications, liver fibrosis, intellectual disability and retinal changes (summarized in Table 1). This boy was short, with hypotonia and dysmorphic facial features. Other than pale skin with darker areas around the eyes, neither skin abnormalities nor dystrophic nails, often observed in patients with DC, were detected (Figure 1A). His hearing and cardiac function appeared normal.

At 21 months of age the proband required transfusions once every other week, due to his thrombocytopenia and erythropenia. Hematopoietic stem cell transplantation was counterindicated by the severity of his disease and, instead, he received androgen therapy. Six years old at the time of this study, he was refractory to infusion of thrombocytes, which he only received when bleeding. He suffered several life-threatening esophageal bleedings. The proband had no speech but communicated with signs and was fed through a gastric tube.

### **Genetic analysis and identification of mutations in *WRAP53***

Despite his delayed development and dysmorphic features, comparative genomic hybridization (CGH) revealed normal copy numbers of chromosomes. In agreement with his HHS-like symptoms, analysis of his peripheral blood leukocytes revealed very short telomeres (below the first percentile in comparison to those of healthy controls of the same age) (Figure 1B). Targeted sequencing of genes known to be related to DC/HHS (i.e., *TERC*, *TINF2* and *DKC1*) did not, however, reveal any mutations. Thus, the proband had classical symptoms of HHS, including extremely short telomeres, but no mutations in genes known to be associated with this disease.

To identify potential underlying mutations, whole-exome sequencing of DNA from the boy and both of his parents was performed. The proband carried two heterozygous missense mutations in the *WRAP53* gene (Gene ID 55135): nucleotides c.847C>T and c.1192C>T, corresponding to p.Leu283Phe (L283F) and p.Arg398Trp (R398W) in the resulting protein (Figure 1C). Heterozygous mutations resulting in R398W has been detected previously in one case of DC, but then in combination with a F164L mutation ^14^, while L283F has not been reported earlier.

The predicted structure of the WRAP53β protein includes an unstructured N-terminal region (including a proline-rich section), a domain with seven WD40 repeats, followed by a short C-terminal extension containing a glycine-rich region (Figure 1C) ^30^. Both L283 and R398 are located within the WD40 domain of WRAP53β and are strictly conserved in all species examined to date, including mammals and flies (Figure 1D).

The healthy father harbored a single *WRAP53* mutation (R398W), but did not demonstrate any manifestations of disease, indicative of autosomal recessive inheritance (Figure 1E). The healthy mother had two wild-type alleles in peripheral blood, indicating that the second mutation in the proband (L283F) had occurred *de novo* (although maternal gonadal mosaicism cannot be excluded). The healthy younger sister of the proband was not tested.

**Mutations in WRAP53β impair its localization to the nucleus and Cajal bodies as well as its structural functions in Cajal body**

To explore how the L283F and R398W changes might influence the behavior of the WRAP53β protein, these mutations were individually incorporated into a vector encoding EGFP-WRAP53β that was then expressed transiently in HeLa cells. Western blotting of whole cell lysates revealed lower levels of both mutant proteins than of the wild-type transfected WRAP53β (Figure 2A). In addition, the RNA levels of L283F, but not R398W, were also reduced (Figure 2B), indicating that the stability of both WRAP53β RNA (at least for L283F) and the corresponding protein are reduced by these mutations. The efficiency of transfection (40-60% of cells, data not shown) was similar in all cases and thus could not explain the differences observed.

To examine whether accelerated proteasomal degradation of the WRAP53β mutants could be an underlying cause of their lower levels, cells expressing these proteins were treated with MG132, a proteasome inhibitor for 8 h before the protein levels were measured. Although both mutants showed a slightly higher stabilization following MG132 compared to wild-type protein (1.2-fold for L283F, 1.5-fold for R398W and 1.1-fold for wild-type), their levels were not restored to those of the wild-type WRAP53β (Figure 2C-D). Together, this indicate that RNA destabilization is the main cause of the lower L283F protein levels, while in the case of R398W, whose RNA levels were normal, an increased rate of protein degradation in combination with a yet unknown factor appear to underlie the reduced protein levels.

Examination by immunofluorescence revealed that the wild-type WRAP53β protein localizes in the cytoplasm and nucleus and is enriched in nuclear Cajal bodies, as reported previously ^14,24,26^. In contrast, the mutant forms were localized to a lesser extent in the nucleus and absent from the Cajal bodies (Figure 2E-G).

Proper assembly of WRAP53β in Cajal bodies is essential for their maintenance, as demonstrated by the findings that either loss or overexpression of WRAP53β causes these organelles to collapse, after which they cannot re-form ^24,26,31,32^. Accordingly, overexpression of exogenous wild-type WRAP53β here led to the disappearance and/or prevented the formation of Cajal bodies in approximately half of the cells transfected (Figure 2H). Interestingly, overexpression of the mutant forms of WRAP53β or of GFP itself produced no such effect (Figure 2H), indicating that the structural function of WRAP53β in connection with Cajal bodies is attenuated by the mutations associated with HHS, probably due to the inability of the mutant proteins to localize in these organelles. Together, these findings demonstrate that the mutations in WRAP53β associated with HHS render the protein unstable, cause mislocalization of the protein, and perturb its structural functions in Cajal bodies.

**Mislocalization of mutant WRAP53β is due to alterations in their interactions with components of the Cajal body and TRiC chaperonin complex**

Localization of WRAP53β to Cajal bodies requires its interaction with coilin and SMN ^26^. While immunoprecipitation of wild-type WRAP53β efficiently co-precipitated both of these proteins, neither WRAP53β L283F nor R398W did (Figure 3A-B), indicating that loss of interaction with coilin and SMN underlies the attenuated accumulation of mutant WRAP53β in Cajal bodies.

Impaired folding of these mutant proteins by the TRiC chaperonin complex might also contribute to their mislocalization, as well as to reduced stability, as observed previously for DC mutants of WRAP53β ^28^. Immunoprecipitation, revealed an enhanced interaction of both mutant forms of WRAP53β with the TRiC chaperonin component CCT6A in comparison to the wild-type protein (Figure 3A-B). Since WRAP53β is only released from TRiC upon proper folding ^28^, it appears like the folding of the mutants is impaired. In addition, this enhanced interaction sequesters WRAP53β mutants in the cytoplasm, preventing its entry into the nucleus (Figure 2E). We conclude that HHS-associated mutations in WRAP53β cause misfolding of the protein and disrupt its interactions with SMN and coilin, thereby preventing its entry into the nucleus and localization in Cajal bodies.

**Mutations in WRAP53β disrupt its interaction with components of telomerase and scaRNAs**

In addition to its structural role in Cajal bodies, WRAP53β targets telomerase and scaRNAs to these organelles. Immunoprecipitation of GFP-WRAP53β followed by western blotting or RT-PCR confirmed that the wild-type protein co-precipitates with components of the telomerase complex (including dyskerin, NHP2, NOP10, GAR1 and TERC) (Figure 3A-B), as well as with various scaRNAs (including those with C/D boxes (i.e., scaRNA2) and H/ACA boxes (i.e., scaRNA5 and TERC)) (Figure 3C). In contrast, neither HHS-mutant of WRAP53β co-precipitated telomerase components or scaRNAs (Figure 3A-C). The fact that disruption of the WRAP53β-TERC interaction prevents the targeting of telomerase to telomeres provides a mechanistic explanation for the severely reduced telomere length in our proband (Figure 1B).

The loss of binding between mutant WRAP53β and components of Cajal bodies and telomerase was almost complete and greater than the reduced expression of the mutants (Figure 3B). Moreover, interaction was also lost under conditions when the levels of the mutants were similar as wild-type (data not shown). Furthermore, the enhanced interaction between mutant WRAP53β and CCT6A demonstrates that the extent of interaction does not necessarily reflect the level of the protein.

**HHS-associated mutations abrogate the involvement of WRAP53β in repair of DNA double-strand breaks**

The mutant forms of WRAP53β retained the capacity to bind the DNA repair proteins RNF8, MDC1 and γH2AX, although somewhat less extensively, particularly in the case of γH2AX (Figure 4A-B). This finding is similar to previous observations on the DC-associated mutants of WRAP53β (F164L, H376Y, R398W and G435R) ^29,33^. We conclude that interactions between WRAP53β and DNA repair proteins are less dependent on structure/folding of WRAP53β than its interaction with Cajal body factors and components of telomerase, but may, instead, depend on the linear sequence.

When DNA damage occurs, WRAP53β targets the repair factor RNF8 to the lesions and facilitates subsequent recruitment of downstream repair factors, including 53BP1 ^29,34^. Following siRNA depletion of the endogenous wild-type WRAP53β the assembly of 53BP1 in repair foci is reduced and we next explored whether HHS mutants of WRAP53β could reverse this loss of 53BP1 assembly on damaged chromatin. When constructs encoding siRNA-resistant forms of wild-type, L283F or R398W WRAP53β were introduced into cells depleted of the endogenous protein, only the wild-type could restore the formation of 53BP1 foci following induction of DNA damage by IR (Figure 4C-D).

The presence of residual γH2AX foci 24 h after damaging DNA with IR is a sign of defective repair. As observed in connection with the formation of repair foci, re-introduction of the L283F or R398W mutant following knockdown of endogenous WRAP53β could not resolve residual γH2AX foci, whereas the wild-type protein did (Figure 4E).

In summary, our findings demonstrate that HHS-linked mutations in *WRAP53* lead to destabilization, mislocalization and impairment of its function in Cajal bodies, at telomeres and in DNA repair. These combined deficiencies contribute to the multiple symptoms and severity of HHS (Figure 5).

**Discussion**

Here, we show for the first time that biallelic mutations in *WRAP53* result in HHS. The missense mutations identified (L283F and R398W) are both located in the WD40 scaffolding domain of the WRAP53β protein and affect amino acids that are highly conserved across species. By scaffolding appropriate interactions, WRAP53β plays essential roles in localizing various factors to the nuclear organelles known as Cajal bodies, to telomeres and to DNA double-strand breaks and is thus important in connection with the regulation of nuclear architecture, telomere elongation and DNA repair ^24,26,29,31^.

Although the explanation for the wide range in severity of DC (including development of HHS) and age of onset remain unclear, the specific genes involved and the mode of inheritance provide part of the answer. In the case of WRAP53β, inherited biallelic mutations affecting its WD40 domain have previously been reported in three patients and linked to DC, including the R398W mutation ^14,22,27^. All patients exhibited at early ages severe symptoms of classic DC, including bone marrow failure, very short telomeres and the triad of skin pigmentation, oral leukoplakia and nail dysplasia. The 30-year old male reported by Shao et al 2018 had short stature, liver cirrhosis, portal hypertension, splenomegaly, marked decreased platelet levels, esophageal varices, and recurrent gastrointestinal hemorrhages, and the 13-year old male reported by Zhong and Batista et al 2011 was diagnosed with esophageal web and narrowing, fusion of the kidneys and pervasive developmental disorder. It is possible that certain mutations, including the novel L283F mutation described here, are more detrimental than others. Indeed, mRNAs containing the c.847C>T mutation (encoding L283F) are expressed at much lower levels than those containing c.1192C>T (encoding R398W), indicating RNA destabilization by the former. However, the functional consequences of L283F and R398W, as examined here, were similar in both cases.

WRAP53β is normally present in the cytoplasm and, at the same time highly enriched in nuclear Cajal bodies. We demonstrate here that the L283F and R398W mutants of WRAP53β are unstable, cannot enter the nucleus properly and do not accumulate in Cajal bodies. Our finding that the interaction between the mutant forms of WRAP53β and the cytoplasmic TRiC chaperonin complex is enhanced, indicates that these mutant proteins are misfolded and therefore cannot be released from this chaperone. These mutants are also unable to bind coilin and SMN. Since TRiC-mediated folding and binding to coilin and SMN are required for accumulation of WRAP53β in Cajal bodies, these observations explain why accumulation of the mutant proteins does not occur ^26,28^.

The inability of mutant WRAP53β to accumulate in Cajal bodies and/or bind known interaction partners prevented various functions of this protein at this site. First, high overexpression of the mutant forms did not result in the collapse of Cajal bodies and/or inability of this organelle to form, in contrast to high overexpression of the wild-type protein ^26,32^. Secondly, the mutant proteins no longer interacted with SMN, coilin, scaRNAs or telomerase, interactions known to be required for the localization of these factors to Cajal bodies ^24-26^. In agreement with such impaired trafficking of telomerase, the telomeres in the proband with HHS were extremely short.

Our proband exhibited a variety of neurological abnormalities, including microcephaly, cerebellar hypoplasia, poor myelinization, delayed psychomotor development, and broad gait. Indeed, underdevelopment of the brain is a key clinical manifestation of HHS and a requirement for definitive diagnosis ^35^. Interestingly, loss of WRAP53β-mediated trafficking of SMN has been observed in patients with spinal muscular atrophy ^26^, a severe neurodegenerative disorder characterized by progressive degeneration of α-motor neurons in the spinal cord, resulting in paralysis and in severe cases, death. Accordingly, depletion of WRAP53β in *Drosophila* and *Caenorhabditis elegance* resulted in defective locomotion and the death of motor neurons, consistent with the spinal muscular atrophy elicited by depletion of SMN ^36^. Mechanistically, expression of SMN in the WRAP53β-deficient cells was reduced and restoration of the level of SMN reversed the defects in locomotion in both species. Importantly, overexpression of WRAP53β also eliminated the defects in locomotion linked to SMN-deficiency in both *Drosophila* and *Caenorhabditis elegance*, demonstrating conserved collaboration between WRAP53β and SMN in this context. Thus, unrelated to telomeres, defective WRAP53β-SMN interplay may cause the neurological complications of WRAP53β-associated HHS. Furthermore, localization of SMN and scaRNAs in Cajal bodies is important for modification and maturation of spliceosomal small nuclear RNAs and dysfunction of this process and, ultimately, of splicing could contribute to the diverse clinical manifestations of HHS and DC.

To date, little evidence links defects in DNA repair to HHS. With the exception of the RTEL1 helicase that suppresses homologous recombination ^37-39^, none of the other proteins encoded by HHS-related genes (*DKC1, TERT, ACD, PARN* and *TINF2)* has been directly implicated in DNA repair. In contrast, WRAP53β has an established role in the repair of DNA double-strand breaks, scaffolding interactions between the repair factors MDC1 and RNF8 at these lesions and thereby promoting subsequent repair by homologous recombination and non-homologous end-joining ^29,32,40^. Our current data reveal that although HHS mutations in WRAP53β reduce its interactions with MDC1, RNF8 and γH2AX to only a moderate extent, they significantly impair assembly of the downstream 53BP1 repair factor at DNA lesions, attenuate the repair of IR-induced damage, and result in accumulation of DNA damage.

Interestingly, WRAP53β organizes and resolves persistent DNA damage in neurons ^41^, such accumulation of damage is believed to contribute to neurodegeneration, again indicating a telomeric-independent function of WRAP53β in connection with brain homeostasis. Together, these results indicate that WRAP53β-mediated defects in DNA repair in the brain and in other tissues may represent a novel contribution to the severity of HHS and DC in general.

In summary, our present findings reveal that compound heterozygous mutations in *WRAP53* cause HHS by disrupting telomere elongation, DNA repair and functions associated with SMN. These combined deficiencies may explain the complex manifestations of this disease in patients. Our observations also highlight the potential contributions of defects in DNA repair and SMN to other cases of HHS and DC cases, where specific underlying mechanisms have yet to be fully elucidated.

**Materials and Methods**

**Inclusion of the patient and ethical considerations**

The patient was referred to the Department of Clinical Genetics, Karolinska University Hospital in Stockholm, Sweden. This study was performed in accordance with the Declaration of Helsinki and pre-approved by the local ethical board in Stockholm. Informed consent was obtained from the proband and his parents in accordance with local ethical guidelines, including the publication of proband photos.

**Array comparative genomic hybridization**

To detect variations in copy number, the 180K custom array (Oxford Gene Technology, Oxford, UK) and a 244k catalogue array (Agilent Technologies, Santa Clara, USA) was utilized. All experiments were performed in accordance with the manufacturer's recommendations, with minor modifications only. Scanning and the computational procedures have been described previously ^42^.

### **Sequence analysis genes known to be related to DC**

Targeted sequencing of *TERC*, *TINF2* (exon 6) and *DKC1* (exon 3, 4 and 11) was performed at Queen Mary University (London).

**Whole-exome capture and resequencing**

DNA from the proband and both parents, isolated from peripheral blood employing standard procedures, was sent for exome sequencing by Oxford Gene Technology, (OGT; Begbroke Science Park, Oxfordshire, UK). These samples were subjected to exome capturing using Agilent SureSelect All Exon (v4). Whole-exome sequencing (WES) was performed on an Illumina HiSeq 2500 instrument and 20x coverage was achieved for 90% of the targeted sequence. These sequences were mapped and compared to the reference sequence (UCSC hg 19) in the published human genome. Data were analyzed with software provided by Oxford Gene Technology. Variants detected were selected for further analysis on the basis of *de novo* and autosomal recessive (homozygous and compound heterozygous) inheritance. The presence of suspected pathogenic variants was verified by Sanger sequencing (primers and PCR conditions available on request).

**Determining telomere length**

DNA extracted from peripheral blood leukocytes was analyzed for relative telomere length (RTL) by quantitative PCR ^43^, with minor modifications as described earlier ^44^. In brief, triplicates were subjected to separate telomere (T) and single copy gene (S) reactions in an ABI 7900HT instrument (Applied Biosystems) on two separate occasions. T/S values were calculated as 2^−ΔCt^, where ΔCt = Ct_(T)_-Ct_(S)_. The RTL value was obtained by dividing the sample T/S value by the T/S of a reference DNA from a cell line (CCRF-CEM) included in all runs. The RTL value of the sample was compared to that of 173 normal controls (age 0-84 years). The generalized additive model included in the GAMLSS R package was used to calculate centile curves. A best-fit model was obtained by applying the Generalised Akaike information criterion, which revealed the Box-Cox Cole and Green distribution to be optimal. “Very short” telomeres were defined as these shorter than the first percentile of the age-matched normal controls.

**Cell lines, culture conditions and transfections.**

HeLa cells (ATCC) were cultured in low-glucose Dulbecco's modified Eagle medium (Gibco, ThermoFisher Scientific) supplemented with 10% fetal bovine serum (Gibco, ThermoFisher Scientific) and 1% Penicillin/Streptomycin (Gibco, ThermoFisher Scientific) at 37 °C in a humidified incubator under 5% CO_2_. Plasmid transfections were performed using Lipofectamine 2000 (Life Technologies, ThermoFisher Scientific) in accordance with the manufacturer’s recommendations and utilizing GFP-Empty, GFP-WRAP53β, GFP-WRAP53β_L283F, GFP-WRAP53β_R398W and Flag-RNF8 expression vectors. Mutations in the WRAP53β plasmids were generated with the QuikChange II XL Site-Directed Mutagenesis kit (Agilent) using the following primers: WRAP53_L283F_For: GGCAGCCCATTCGTTCTGCTTCTCCCC, WRAP53_L283F_Rev: GGGGAGAAGCAGAACGAATGGGCTGCC, WRAP53_R398W_For: CTGTGCTGGGATCTCTGGCAGTCTGGTTACC, WRAP53_R398W_Rev: GGTAACCAGACTGCCAGAGATCCCAGCACAG, WRAP53_siW2res_For: GCAAACGGGAGTCTCTCTGAAGAAGAAGC, WRAP53_siW2res_Rev: GCTTCTTCTTCAGAGAGACTCCCGTTTGC. siRNA (15 nM) targeting WRAP53β (SI00388948, Qiagen) or the scramble control (1027280, Qiagen) were transfected into the cells for 48 h employing the HiPerfect transfection reagent (Qiagen) in accordance with the manufacturer’s recommendations. The protease inhibitor MG132 (Merck) was used at a concentration of 10μM for 8 h.

**Ionizing radiation**

γ-Irradiation was performed with the X-Ray Irradiator CIX2 (X-strahl) at settings of 195 kV and 10 mA, a focus-to-specimen distance (FSD) of 40 cm, and a 3 mm Aluminum filter. The dose was approximately 1.3 Gy/min.

**Immunofluorescent microscopy**

Cells grown on sterilized cover slips were fixed with 4% paraformaldehyde for 15 min at room temperature; permeabilized with 0.1% Triton X-100 for 5 min at room temperature; and then incubated for one hour in blocking buffer (2% BSA, 5% glycerol, 0.2% Tween20, 0.1% NaN3). The cover slips were subsequently incubated with primary antibodies for one hour at room temperature, followed by 30 min with the secondary antibody, both diluted in blocking buffer. After mounting with Vectashield medium containing DAPI (Vector Laboratories, Bionordika), images were acquired with an LSM700 confocal microscope (Zeiss) mounted on a Zeiss Axio observer.Z1 equipped with Plan-Apochromat × 63/1.4 oil immersion lenses and subsequently processed utilizing the Zen 2012 Black software (Zeiss).

**Western blotting**

Cells were harvested, washed and lysed in ice-cold lysis buffer (50 mM Tris-HCL, pH 8, 150 mM NaCl, 1% NP-40, 1% protease inhibitor cocktail) for 30 min on ice. The lysates were cleared by centrifugation at 14 000 rpm for 10 min at 4 °C and the protein concentration determined with the Bradford assay (Bio-Rad). Proteins were resolved on 10% NuPAGE^®^ Bis-Tris or 3-8% Tris-Acetate precast gels (Life Technologies, ThermoFisher Scientific) and transferred onto nitrocellulose membranes. Western blotting was performed by standard procedures and the blots developed using SuperSignal West Femto Maximum Sensitivity Substrate (ThermoFisher Scientific). Protein levels were quantified by image densitometry of western blot images using ImageJ.

**Antibodies**

The following antibodies against the human proteins were utilized for immunofluorescent staining and western blotting: rabbit α-CCT6A (HPA045576, Atlas Antibodies), mouse α-coilin (sc-56298, Santa Cruz Biotechnology), rabbit α-coilin (sc-32860, Santa Cruz Biotechnology), rabbit α-DKC1 (sc-48794, Santa Cruz Biotechnology), mouse α-GAPDH (sc-47724, Santa Cruz Biotechnology), rabbit α-GAR1 (11711-1-AP, ProteinTech), rabbit α-GFP (ab290, Abcam), rabbit α-IgG (12-370, Millipore, Merck), mouse α-MDC1 (ab50003, Abcam), mouse α-NHP2 (sc-398430 ,Santa Cruz Biotechnology), rabbit α-NOP10 (ab134902, Abcam), mouse α-RNF8 (sc-271462, Santa Cruz Biotechnology), mouse α-SMN (610647, BD Biosciences), rabbit α-53BP1 (NB100- 904, Novus Biologicals, Bio-Techne), mouse α-γH2AX (05-636, Millipore), rabbit α-γH2AX (2577, Cell Signaling, Bionordika). The secondary antibodies used were: goat α-rabbit HRP-conjugated (cat. no. 7074, Cell Signaling), horse α-mouse HRP-conjugated (cat. no. 7076, Cell Signaling), donkey α-mouse Alexa Fluor 594 (cat. no. A21203, Invitrogen) and donkey α-rabbit Alexa Fluor 594 (cat. no. A21207, Invitrogen).

**Immunoprecipitation**

Cells were washed twice with ice-cold PBS, scraped of the plates and pelleted by centrifugation. The pellet thus obtained was treated with ice-cold lysis buffer (50 mM Tris-HCL, pH 8, 150 mM NaCl, 1% NP-40, 1% protease inhibitor cocktail) for 15 min on ice, followed by sonication at high intensity for 5 min (30 sec ON/30 sec OFF) in a Bioruptor UCD-200 (Diagenode). The resulting lysate was then centrifuged at 6000 rpm for 5 min at 4 °C and 1 mg supernatant protein incubated with 10 μl Dynabeads^TM^ Protein G (Invitrogen) and 1 μg GFP antibody overnight at 4 °C with rotation. Thereafter, the beads were washed 4 times with lysis buffer and prepared for western blotting.

**RNA Immunoprecipitation**

Cell lysates were prepared for overnight immunoprecipitation as described above. The beads were washed twice in lysis buffer and then twice in RIPA buffer. RNA was extracted with TRIzol^®^ (Life Technologies, ThermoFisher Scientific), 1-bromo-3-chloropropane (Sigma-Aldrich) and the RNeasy Mini Kit (Qiagen), following the manufacturer’s instructions.

**qPCR analysis**

cDNA was generated with SuperScript IV reverse transcriptase (Invitrogen, ThermoFisher Scientific), random hexamer primers (ThermoFisher Scientific), 10 nM dNTPs Mix (ThermoFisher Scientific) and RNaseOUT (Invitrogen, ThermoFisher Scientific) in saccordance with manufacturer’s instructions. Enrichment of specific RNA was determined by qPCR in a 7500 Fast Real-Time PCR System (Applied Biosystems, ThermoFisher Scientific) on 96-well fast PCR Plates (Sarstedt) using Fast SYBR™ Green Master Mix (Applied Biosystems, ThermoFisher Scientific) and the following primers, TERC For: GTGGTGGCCATTTTTTGTCTAAC, TERC Rev: TGCTCTAGAATGAACGGTGGAA, scaRNA2 For: GGTTGGAGCGTGTTAGGC, scaRNA2 Rev: GGAGGAGACCTTTTCATTTCG, scaRNA5 For: TGAATGTCACGGTCCCTTTGT, scaRNA5 Rev: AGCTGCTCCATGATCCCATAC , β-actin For: AGAGCTACGAGCTGCCTGAC, β-actin Rev: AGCACTGTGTTGGCGTACAG, GFP For: CTCCTGCCCGACAACCAC , GFP Rev: TCACGAACTCCAGCAGGAC.

**Quantification and statistical analysis**

Quantitative immunofluorescence was performed in at least 3 independent experiments. Statistical details and the numbers of cells analyzed are indicated in the figure legends. Statistical analysis with non- paired two-tailed Student’s t-tests was carried out in the Microsoft Excel 16.16.12 software.

**Conflicts of interest**

The authors have no conflicting financial interests relevant to this article.

**Acknowledgements**

We would like to thank the boy and his parents for their participation. This work was supported by grants from the Swedish Childhood Cancer Foundation, the Swedish Cancer Society, the Center for Innovative Medicine, the Strategic Research Program in Cancer, the Cancer Society of Stockholm, the Swedish Society for Medical Research, the Swedish Research Council, Berth von Kantzow’s Foundation, Karolinska Institutet, the Stockholm County Council, the Swedish Brain Foundation and The Hållsten Research Foundation.

**References**

1 Niewisch, M. R. & Savage, S. A. An update on the biology and management of dyskeratosis congenita and related telomere biology disorders. *Expert Rev Hematol*, 1-16, doi:10.1080/17474086.2019.1662720 (2019).

2 Savage, S. A. Beginning at the ends: telomeres and human disease. *F1000Res* **7**, doi:10.12688/f1000research.14068.1 (2018).

3 Heiss, N. S. *et al.* X-linked dyskeratosis congenita is caused by mutations in a highly conserved gene with putative nucleolar functions. *Nat Genet* **19**, 32-38, doi:10.1038/ng0598-32 (1998).

4 Knight, S. W. *et al.* Unexplained aplastic anaemia, immunodeficiency, and cerebellar hypoplasia (Hoyeraal-Hreidarsson syndrome) due to mutations in the dyskeratosis congenita gene, DKC1. *Br J Haematol* **107**, 335-339, doi:10.1046/j.1365-2141.1999.01690.x (1999).

5 Yaghmai, R. *et al.* Overlap of dyskeratosis congenita with the Hoyeraal-Hreidarsson syndrome. *J Pediatr* **136**, 390-393, doi:10.1067/mpd.2000.104295 (2000).

6 Marrone, A. *et al.* Telomerase reverse-transcriptase homozygous mutations in autosomal recessive dyskeratosis congenita and Hoyeraal-Hreidarsson syndrome. *Blood* **110**, 4198-4205, doi:10.1182/blood-2006-12-062851 (2007).

7 Ballew, B. J. *et al.* Germline mutations of regulator of telomere elongation helicase 1, RTEL1, in Dyskeratosis congenita. *Hum Genet* **132**, 473-480, doi:10.1007/s00439-013-1265-8 (2013).

8 Deng, Z. *et al.* Inherited mutations in the helicase RTEL1 cause telomere dysfunction and Hoyeraal-Hreidarsson syndrome. *Proc Natl Acad Sci U S A* **110**, E3408-3416, doi:10.1073/pnas.1300600110 (2013).

9 Walne, A. J., Vulliamy, T., Kirwan, M., Plagnol, V. & Dokal, I. Constitutional mutations in RTEL1 cause severe dyskeratosis congenita. *Am J Hum Genet* **92**, 448-453, doi:10.1016/j.ajhg.2013.02.001 (2013).

10 Le Guen, T. *et al.* Human RTEL1 deficiency causes Hoyeraal-Hreidarsson syndrome with short telomeres and genome instability. *Hum Mol Genet* **22**, 3239-3249, doi:10.1093/hmg/ddt178 (2013).

11 Tummala, H. *et al.* Poly(A)-specific ribonuclease deficiency impacts telomere biology and causes dyskeratosis congenita. *J Clin Invest* **125**, 2151-2160, doi:10.1172/jci78963 (2015).

12 Savage, S. A. *et al.* TINF2, a component of the shelterin telomere protection complex, is mutated in dyskeratosis congenita. *Am J Hum Genet* **82**, 501-509, doi:10.1016/j.ajhg.2007.10.004 (2008).

13 Vulliamy, T. *et al.* The RNA component of telomerase is mutated in autosomal dominant dyskeratosis congenita. *Nature* **413**, 432-435, doi:10.1038/35096585 (2001).

14 Zhong, F. *et al.* Disruption of telomerase trafficking by TCAB1 mutation causes dyskeratosis congenita. *Genes Dev* **25**, 11-16, doi:10.1101/gad.2006411 (2011).

15 Walne, A. J. *et al.* Genetic heterogeneity in autosomal recessive dyskeratosis congenita with one subtype due to mutations in the telomerase-associated protein NOP10. *Hum Mol Genet* **16**, 1619-1629, doi:10.1093/hmg/ddm111 (2007).

16 Vulliamy, T. *et al.* Mutations in the telomerase component NHP2 cause the premature ageing syndrome dyskeratosis congenita. *Proc Natl Acad Sci U S A* **105**, 8073-8078, doi:10.1073/pnas.0800042105 (2008).

17 Keller, R. B. *et al.* CTC1 Mutations in a patient with dyskeratosis congenita. *Pediatr Blood Cancer* **59**, 311-314, doi:10.1002/pbc.24193 (2012).

18 Kocak, H. *et al.* Hoyeraal-Hreidarsson syndrome caused by a germline mutation in the TEL patch of the telomere protein TPP1. *Genes Dev* **28**, 2090-2102, doi:10.1101/gad.248567.114 (2014).

19 Vulliamy, T. J. *et al.* Differences in disease severity but similar telomere lengths in genetic subgroups of patients with telomerase and shelterin mutations. *PLoS One* **6**, e24383, doi:10.1371/journal.pone.0024383 (2011).

20 Walne, A. J., Vulliamy, T., Beswick, R., Kirwan, M. & Dokal, I. Mutations in C16orf57 and normal-length telomeres unify a subset of patients with dyskeratosis congenita, poikiloderma with neutropenia and Rothmund-Thomson syndrome. *Human molecular genetics* **19**, 4453-4461, doi:10.1093/hmg/ddq371 (2010).

21 Touzot, F. *et al.* Heterogeneous telomere defects in patients with severe forms of dyskeratosis congenita. *J Allergy Clin Immunol* **129**, 473-482, 482.e471-473, doi:10.1016/j.jaci.2011.09.043 (2012).

22 Batista, L. F. *et al.* Telomere shortening and loss of self-renewal in dyskeratosis congenita induced pluripotent stem cells. *Nature* **474**, 399-402, doi:10.1038/nature10084 (2011).

23 Mahmoudi, S. *et al.* Wrap53, a natural p53 antisense transcript required for p53 induction upon DNA damage. *Molecular cell* **33**, 462-471, doi:10.1016/j.molcel.2009.01.028 (2009).

24 Venteicher, A. S. *et al.* A human telomerase holoenzyme protein required for Cajal body localization and telomere synthesis. *Science* **323**, 644-648, doi:10.1126/science.1165357 (2009).

25 Tycowski, K. T., Shu, M. D., Kukoyi, A. & Steitz, J. A. A conserved WD40 protein binds the Cajal body localization signal of scaRNP particles. *Mol Cell* **34**, 47-57, doi:10.1016/j.molcel.2009.02.020 (2009).

26 Mahmoudi, S. *et al.* WRAP53 is essential for Cajal body formation and for targeting the survival of motor neuron complex to Cajal bodies. *PLoS biology* **8**, e1000521, doi:10.1371/journal.pbio.1000521 (2010).

27 Shao, Y. *et al.* A unique homozygous WRAP53 Arg298Trp mutation underlies dyskeratosis congenita in a Chinese Han family. *BMC Med Genet* **19**, 40, doi:10.1186/s12881-018-0549-1 (2018).

28 Freund, A. *et al.* Proteostatic control of telomerase function through TRiC-mediated folding of TCAB1. *Cell* **159**, 1389-1403, doi:10.1016/j.cell.2014.10.059 (2014).

29 Henriksson, S. *et al.* The scaffold protein WRAP53beta orchestrates the ubiquitin response critical for DNA double-strand break repair. *Genes Dev* **28**, 2726-2738, doi:10.1101/gad.246546.114 (2014).

30 Bergstrand, S., O'Brien, E. M. & Farnebo, M. The Cajal Body Protein WRAP53beta Prepares the Scene for Repair of DNA Double-Strand Breaks by Regulating Local Ubiquitination. *Front Mol Biosci* **6**, 51, doi:10.3389/fmolb.2019.00051 (2019).

31 Wang, Q. *et al.* Cajal bodies are linked to genome conformation. *Nat Commun* **7**, 10966, doi:10.1038/ncomms10966 (2016).

32 Rassoolzadeh, H. *et al.* Overexpression of the scaffold WD40 protein WRAP53beta enhances the repair of and cell survival from DNA double-strand breaks. *Cell Death Dis* **7**, e2267, doi:10.1038/cddis.2016.172 (2016).

33 Rassoolzadeh, H., Coucoravas, C. & Farnebo, M. The proximity ligation assay reveals that at DNA double-strand breaks WRAP53beta associates with gammaH2AX and controls interactions between RNF8 and MDC1. *Nucleus* **6**, 417-424, doi:10.1080/19491034.2015.1106675 (2015).

34 Hedstrom, E. *et al.* Downregulation of the cancer susceptibility protein WRAP53beta in epithelial ovarian cancer leads to defective DNA repair and poor clinical outcome. *Cell Death Dis* **6**, e1892, doi:10.1038/cddis.2015.250 (2015).

35 Glousker, G., Touzot, F., Revy, P., Tzfati, Y. & Savage, S. A. Unraveling the pathogenesis of Hoyeraal-Hreidarsson syndrome, a complex telomere biology disorder. *Br J Haematol* **170**, 457-471, doi:10.1111/bjh.13442 (2015).

36 Di Giorgio, M. L. *et al.* WDR79/TCAB1 plays a conserved role in the control of locomotion and ameliorates phenotypic defects in SMA models. *Neurobiol Dis* **105**, 42-50, doi:10.1016/j.nbd.2017.05.005 (2017).

37 Barber, L. J. *et al.* RTEL1 maintains genomic stability by suppressing homologous recombination. *Cell* **135**, 261-271, doi:10.1016/j.cell.2008.08.016 (2008).

38 Uringa, E. J., Youds, J. L., Lisaingo, K., Lansdorp, P. M. & Boulton, S. J. RTEL1: an essential helicase for telomere maintenance and the regulation of homologous recombination. *Nucleic Acids Res* **39**, 1647-1655, doi:10.1093/nar/gkq1045 (2011).

39 Recker, J., Knoll, A. & Puchta, H. The Arabidopsis thaliana homolog of the helicase RTEL1 plays multiple roles in preserving genome stability. *Plant Cell* **26**, 4889-4902, doi:10.1105/tpc.114.132472 (2014).

40 Coucoravas, C., Dhanjal, S., Henriksson, S., Bohm, S. & Farnebo, M. Phosphorylation of the Cajal body protein WRAP53beta by ATM promotes its involvement in the DNA damage response. *RNA Biol* **14**, 804-813, doi:10.1080/15476286.2016.1243647 (2017).

41 Mata-Garrido, J., Casafont, I., Tapia, O., Berciano, M. T. & Lafarga, M. Neuronal accumulation of unrepaired DNA in a novel specific chromatin domain: structural, molecular and transcriptional characterization. *Acta Neuropathol Commun* **4**, 41, doi:10.1186/s40478-016-0312-9 (2016).

42 Winberg, J. *et al.* Partial tetrasomy 14 associated with multiple malformations. *Am J Med Genet A* **161A**, 1284-1290, doi:10.1002/ajmg.a.35887 (2013).

43 Cawthon, R. M. Telomere measurement by quantitative PCR. *Nucleic Acids Res* **30**, e47 (2002).

44 Norberg, A. *et al.* Novel variants in Nordic patients referred for genetic testing of telomere-related disorders. *Eur J Hum Genet* **26**, 858-867, doi:10.1038/s41431-018-0112-8 (2018).

Table 1 Characteristics of the proband and his parents

| **Individual** | **Gender** | **Age when the study was performed (yrs)** | **Diagnosis** | **Clinical features/abnormalities** | **Telomere length** | **Genotype** |
| --- | --- | --- | --- | --- | --- | --- |
| Proband | Male | 6 | HHS | Intrauterine growth restriction (IUGH)  **Gastrointestinal complications**  Necrotizing enterocolitis (NEC)  Volvulus (50% of his small intestine was removed)  Digestive tract anomalies and eating difficulties (i.e., oesophageal strictures, varices, swallowing difficulties and severe hemorrhages)  **Neurological complications**  Microcephaly  Cerebellar hypoplasia  Atrophy of the vermis cerebelli  Cortical malformation  Poor myelinization  Delayed psychomotor development (i.e., broad gait, mild intellectual disability, no speech)  **Bone marrow failure and immunodeficiency**  Thrombocytopenia  Erythropenia  Recurrent infections  **Other complications**  Fibrosis (grade 3 out of 4) and malformation of the liver  Dilated intrahepatic choledochus  Elevated blood levels of transaminases  Retinal abnormalities and vitreous body hemorrhaging  Dysmorphic features of the face and body (i.e., strabismus, epicanthal folds, slightly deep-set eyes, dark grey eyes, cup-shaped protruding overfolded ears, depressed nasal tip, fine blond hair, widely spaced teeth, full lips, everted upper lip vermilion, fragile, fast growing nails, short stature and hypotonia)  Pale skin | < 1^st^  percentile | WRAP53^L283F^ /WRAP53^R398W^ |
| Father | Male | 45 | Healthy | None | Not determined | WRAP53^WT^  /WRAP53^R398W^ |
| Mother | Female | 44 | Healthy | None | Not determined | WRAP53^WT^  /WRAP53^WT^ |
