# Supplementary material for: Biallelic mutations in WRAP53 result in dysfunctional telomeres, Cajal bodies and DNA repair, thereby causing Hoyeraal–Hreidarsson syndrome
Source: Cell Death Dis. 2020 Apr 17;11(4):238. doi: 10.1038/s41419-020-2421-4 (PMC7165179; doi:10.1038/s41419-020-2421-4)
Supplement: Supplementary file 2 — Athor contribution [file 41419_2020_2421_MOESM2_ESM.pdf]

# DECLARATION OF CONTRIBUTIONS TO ARTICLE

ADMC

Manuscript Number: CDDIS-19-4153

Journal Name: *Cell Death & Disease* (the 'Journal')

Proposed Title of the Contribution:

Biallelic mutations in WRAP53 result in dysfunctional telomeres, Cajal bodies and DNA repair, thereby causing Hoyeraal-Hreidarsson syndrome (the 'Contribution)

Author(s):

Sofie Bergstrand1, Stefanie Böhm2, Helena Malmgren3,4Anna Norberg5, Mikael Sundin6,7 Ann Nordgren3,4,# and Marianne Farnebo1,2,#, * (the 'Authors')

For all *CDDis* articles, each person named as an author in the published version must be able to show he or she has contributed substantially to the article.

Authorship credit should be based on 1) substantial contributions to conception and design, acquisition of data, or analysis and interpretation of data; 2) drafting the article or revising it critically for important intellectual content; and 3) final approval of the version to be published. Authors should meet conditions 1, 2 and 3.

Any person who cannot be shown to have made a substantial contribution to the article cannot be listed as an author in the final version. The name of any person who is deemed to have made a minor contribution can, however, appear in the Acknowledgments section of the article.

Please complete the table below to indicate the contributions of all named authors to the manuscript.

| Author Full Name: | Specification of Contribution to the Manuscript: |
|---|---|
| Sofie Bergstrand | performed molecular/cellular characterization of the mutants and wrote the manuscript in consultation with all of the other authors |
| Stefanie Böhm | performed molecular/cellular characterization of the mutants |
| Helena Malmgren | performed the genetic and clinical analyses on the proband |
| Anna Norberg | analysed telomere length |
| Mikael Sundin | performed the genetic and clinical analyses on the proband |
| Ann Nordgren | performed the genetic and clinical analyses on the proband |
| Marianne Farnebo | performed molecular/cellular characterization of the mutants and wrote the manuscript in consultation with all of the other authors |
| | |
| | |
| | |
| | |
| | |
| | |

Please complete the table below to indicate the contributions of all named authors to the figures.

Figure 1:

HM, AN, MS, ANorb, SB, MF

Figure 2:

SB, StB, MF

Figure 3:

SB, StB, MF

Figure 4:

SB, StB, MF

Figure 5:

SB, MF

Figure 6:

| Signed for and on behalf of the Author(s): | Print Name: | Date: |
| --- | --- | --- |
| Marianne Farnebo | Marianne Farnebo | 19 February 2020 |
